# Year-Old Babies

**Published:** 1891-04

**Authors:** 


### YEAR-OLD BABIES.

Does a two-year-old baby pay for itself up to the time it reaches that interesting age ? Sometimes I think not. I thought so yesterday when my own baby slipped into my study and "scrubbed" the carpet and his best white dress with my bottle of ink. He was playing in the coal hod ten minutes after a clean dress was put on him, and later in the day he pasted fifty cents' worth of postage stamps on the parlor wall and poured a dollar's worth of the choicest "White Rose" perfumery out of the window "to see it wain."

Then he dug out the centre of a nicely baked loaf of cake, and was found in the middle of the dining-room table with the sugar bowl between his legs and most of the contents in his stomach.

He has already cost over $100 in doctors' bills, and I feel that I am right in attributing my few gray hairs to the misery I endured walking the floor with him at night during the first year of his life.

What has he ever done to pay me for that ?

Ah ! I hear his little feet pattering along out in the hall. I hear his little ripple of laughter because he has escaped from his mother and has found his way up to my study at a forbidden hour. But the door is closed. The worthless little vagabond can't get in, and I won't open it for him. No, I won't. I can't be disturbed when I am writing.

He can just cry if he wants to. I won't be bothered for—" rat, tat, tat," go his dimpled knuckles on the door. I sit in silence.

" Rat, tat, tat."

I sit perfectly still.

" Papa."

No reply.

" Peeze, papa."

Grim silence.

" Baby tum in—peeze, papa."

He shall not come in.

" My papa."

I write on.

" Papa," says the little voice ; " I lub my papa. Peeze let baby in ! "

I am not quite a brute, and I throw open the door. In he comes with outstretched little arms, with shining eyes, with laughing face. I catch him up into my arms, and his warm, soft little arms go around my neck, the not very clean little cheek is laid close to mine, the baby voice says sweetly,—

" I lub my papa."

Does he pay ?

Well, I guess he does ! He has cost me many anxious days and nights. He has cost me time and money and care and self-sacrifice. He may cost me pain and sorrow. He has cost much. But he has paid for it all again and again and again in whispering those three little words into my ears : "I lub papa."

Our children pay when their very first feeble little cries fill our hearts with the mother love and the father love that ought never to fail among all earthly passions.

Do our children pay ?—*J. H. D., in Detroit Free Press.*

